# Electrocatalysis with molecules and molecular assemblies within gas diffusion electrodes

**DOI:** 10.1039/d3sc05362h

**Published:** 2023-11-02

**Authors:** Hossein Bemana, Morgan McKee, Nikolay Kornienko

**Affiliations:** a Department of Chemistry, Université de Montréal 1375 Avenue Thérèse-Lavoie-Roux Montréal QC H2V 0B3 Canada nkornien@uni-bonn.de; b Institute of Inorganic Chemistry, University of Bonn Gerhard-Domagk-Str. 1 53121 Bonn Germany

## Abstract

Molecular catalysts and their assemblies are important model systems in electrocatalysis. This is largely because their active sites, secondary coordination spheres, and reaction environments can be rationally modulated. Such experiments yield important insights into the structure–activity relationships that can be used to design improved catalysts or translated to more technologically mature systems. However, in the context of electrocatalysis, molecular catalysts are often dissolved in an electrolyte or heterogenized on an electrode that is completely submersed in an electrolyte (*e.g.* H-cell) or reaction setups that are not used in practical systems and use poorly soluble gaseous reactants like CO_2_, CO, or O_2_. This is beginning to change, with a growing emphasis being placed on investigating molecular catalysts and catalytic assemblies (*e.g.* metal/covalent organic frameworks and polymers with molecular active sites) in gas-diffusion electrodes (GDEs) that feed the reactant directly from the gas phase to the catalytic sites and enable industrially viable current densities. Against this backdrop, this perspective first details the emerging set of molecular catalyst-embedded GDE-based systems and what the community has learned thus far from these efforts. We next identify the gaps in knowledge and performance that are yet to be closed and offer strategies for exploring in this direction. Finally, we conclude with a forward-looking discussion that highlights several new avenues to be pursued with molecule-based GDE platforms and how this can accelerate progress in the electrocatalysis field as a whole.

## Abstract 2

Molecular catalysts and their assemblies are important model systems in electrocatalysis at gas–liquid–solid interfaces.

## Graphical abstract

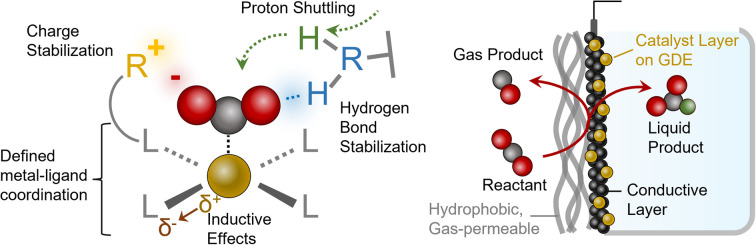

## Introduction

While industrial processes are often catalyzed by heterogeneous catalysts,^1^ molecular catalysts play an important role in growing the fundamental knowledge of the community about these reactions. Although molecular catalysts are used in industries for certain reactions,^2^ work in the area of molecular systems is often aimed at developing key structure–activity relationships that can then be transferred into more robust and technologically mature heterogeneous systems.^3^

**Fig. 1:**
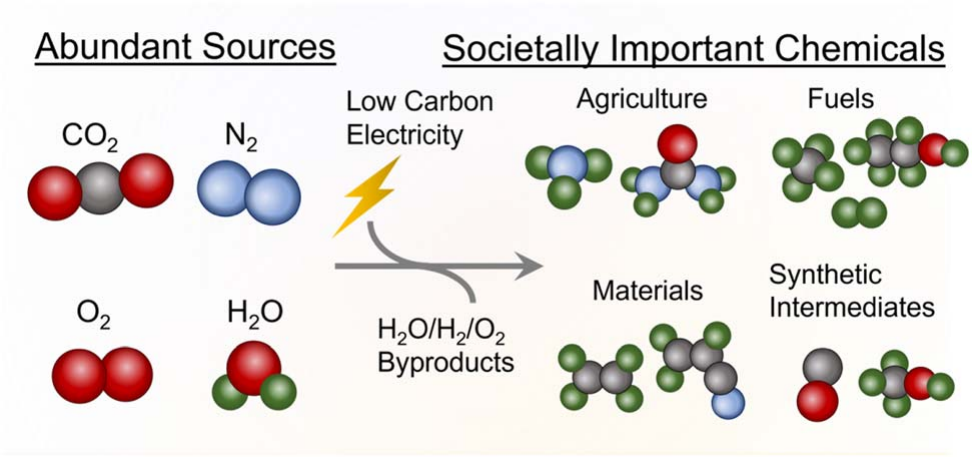
The general concept of electrocatalysis for sustainable chemical production.

To this end, this perspective is focused on an emerging area of high societal importance where fundamental insights derived from molecular catalyst-based systems play a key role. The area of emphasis here is electrocatalytic systems, which have received significant attention in the recent years because they can (ideally) use low-carbon electricity to catalyze reactions that are currently being performed through thermochemical means (Fig. 1). This, in principle, can reduce the carbon footprint of fuel, fertilizer, and commodity chemical production if certain performance metrics of electrochemical systems are attained. For example, the Haber-Bosch synthesis of NH_3_, a highly important agricultural molecule and chemical feedstock, uses up to 1–2% of the world's energy and contributes a considerable amount of CO_2_ emissions.^4^ Using H_2_ from water electrolysis instead of steam reforming can, in theory, lead to 75% less CO_2_ emissions per NH_3_ produced.^5^ A further advantage is that electrochemical systems can obtain many of the most important chemicals to society through abundantly available raw reactants (H_2_O, CO_2_, N_2,_*etc.*) instead of relying on fossil-based feedstocks, which are finite in abundance and concentrated in select areas around the world. Finally, electrochemical systems often operate at (near) ambient conditions and can, therefore, circumvent the need for large-scale infrastructure used for high-temperature/pressure reactions. This naturally opens them up for decentralized applications to yield a product directly where it is needed.

While the aforementioned advantages are appealing, particularly from a sustainability perspective, technologies like CO_2_ reduction (CO_2_R) and N_2_ reduction (NRR) must still mature to attain techno-economic viability and be deployed commercially at scale.^6–8^ One of the key areas for improvement here is the development of more active and selective catalysts. To this end, the development of fundamental insights into structure–activity relationships is a key driver of growth in this area, and this is where research into molecular catalysts can play a substantial role.

**Fig. 2:**
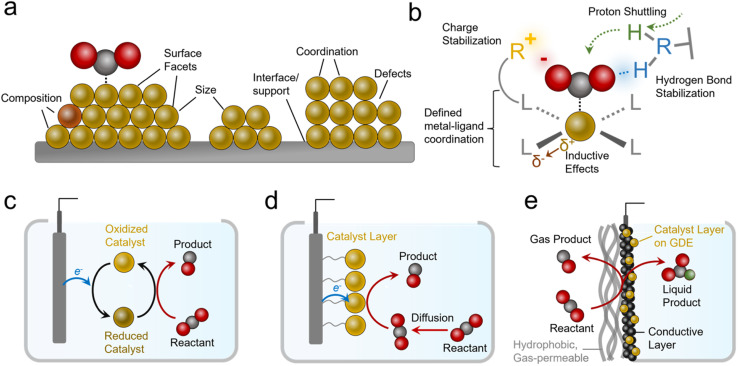
The diversity of active sites within heterogeneous electrocatalysts (a) contrasts with the well-defined catalytic sites and functional environments of molecular systems (b). Molecular catalysts can function as solubilized species (c) or heterogenized on electrode surfaces (d) or within gas-diffusion electrodes (e).

Practical systems that catalyze reactions like the CO_2_R to generate carbon-based products typically feature metal, metal oxide, or nanostructured carbon-based catalysts.^9^ Such materials feature several attractive attributes, namely their high activity (measured in current density, mA cm^−2^), and stability. However, a key limitation in heterogeneous catalysts is the diversity of active sites that are present on their surfaces (Fig. 2a). These include heterogeneity in their size, shape, composition, coordination environment, crystal facets, defects and more.^10^ This inherent heterogeneity renders fundamental studies challenging, especially for studies aiming to modify one variable at a time within a well-defined system, although progress is being made with studies of single-crystal surfaces and atomically precise active sites.^11^

In contrast to the case of heterogeneous systems, molecular electrocatalysts lend themselves quite well to such endeavors because they feature atomically precise metal–ligand coordination (Fig. 2b).^12^ Here, ligands can be systematically modified to electronically influence the metal active sites (or serve as secondary active sites themselves).^13^ In addition, judiciously selected ligands can accelerate catalysis through stabilizing intermediates *via* electrostatic interactions or hydrogen bonds.^14^ Further, they can facilitate reactivity by acting as proton relays.^15,16^ A class of catalyst that features molecular active sites includes metal and covalent organic frameworks (MOFs and COFs),^17^ polymers,^18^ and other molecular assemblies with defined molecular sites embedded within a chemically-defined porous scaffold. MOFs and COFs offer additional knobs to turn since their scaffolds can be functionalized to generate hydrophobic/hydrophilic environments, shuttle/filter chemical species, and stabilize reaction intermediates.^19,20^ Single-atom catalysts (SACs), systems that feature transition metals embedded within graphene sheets with precise coordination to N/C/O atoms, and their analogues are also an important class of molecular-like electrocatalytic systems. While we will touch on this subject, their comprehensive analysis is beyond the scope of this perspective and we can point interested readers to more focused reviews in this area.^21–23^

Molecular electrocatalysts are often evaluated as dissolved species in an electrolyte with the reactant (*e.g.* CO_2_) also dissolved (Fig. 2c). Once they are oxidized/reduced, they can undergo a further series of chemical reactions with the reactant and/or further electrochemical steps until they are once again regenerated and ready for a subsequent catalytic cycle.^12^ Such systems lend themselves to fundamental studies^24^ but are more removed from practical heterogeneous electrocatalytic systems. Systems that partially bridge this gap are ‘heterogenized’ molecular electrocatalysts (Fig. 2d).^25^ These entail molecules adsorbed, grafted, or grown on heterogeneous electrodes,^13^ as well as MOFs/COFs deposited/grown on electrode surfaces.^26–28^ Heterogenized molecular systems have advantages since their active sites are ‘wired’ to the electrode the entire time while still being accessible to reactants in the electrolyte, a situation more closely resembling heterogeneous material electrocatalysts. One key additional parameter is the link between the catalyst and electrode, which plays a determining role in stability (minimizing desorption) and activity (facilitating electron transfer).

Heterogenized molecular systems can attain high rates for reactions like water reduction/oxidation because the reactant is present in high concentrations. In contrast, for reactions involving gas-phase reactants like the CO_2_R, NRR, and oxygen reduction reaction (ORR), reactants are poorly soluble in aqueous electrolytes, and this limits reaction rates due to mass transport constraints. Therefore simple ‘heterogenized’ catalysts applied in H-cells still feature a gap with practical electrochemical systems that utilize gas diffusion electrode (GDE) geometries that bring in reactants directly from the gas phase to the active sites (Fig. 2e).^29,30^ While the details can vary (see reactor configuration section), such electrodes have one side open to a stream of gas-phase reactants fed directly to the electrode instead of being solubilized in an electrolyte beforehand. These electrodes typically feature a gas-permeable hydrophobic support on top of which sits a catalyst layer like a metal film or a microporous carbon layer coated with a catalyst layer that is exposed to the electrolyte. Alternatively, the catalyst layer can be attached directly to an ion exchange membrane instead of facing the electrolyte in a membrane electrode assembly (MEA). The primary advantage of GDEs is that industrially relevant current densities can be attained^31^ and commercial systems for reactions like the CO_2_R could incorporate them. As a general note, industrially viable current densities are above 200 mA cm^−2^ for most reactions.^8,32^ In fact, such geometries are used in H_2_/O_2_ fuel cells that rely on gaseous reactants.^33^ Further, alkaline electrolytes that suppress the hydrogen evolution reaction (HER) but feature even lower CO_2_ solubility can be used in these geometries (though carbonate formation is a challenge^34^).

In GDEs, molecular catalysts are beginning to be investigated, yielding an array of exciting results (Table 1). Advances in strategies for the fabrication and implementation of molecule-based electrodes have even yielded several systems with comparable performance metrics to noble metal-based catalysts.^35^ An evaluation of these studies, as well as a forward-looking evaluation of this emerging topic, is the focus of this perspective. While the CO_2_R will be the reaction most discussed due to the largest body of work thus far, we will also touch on CO reduction (COR), ORR, and the hydrogen oxidation reaction (HOR) as areas where this paradigm was extended.

**Table 1:** Molecular CO_2_R/COR catalysts discussed in this work and various metrics of their performance. Characterization methods (if available) of catalysts post-electrolysis are listed

Catalyst	Products	FE (%)	*j* (mA cm^−2^)	Stability	Post-catalysis characterization	Electrolyte	Reactor	Ref.
CoPc-CN molecules supported on CNTs	CO from CO_2_	94	82	10 h 2.0 V, near 100% FE, *j* retention	—	1 M KOH	Flow cell	59
CoPc	CO from CO_2_	>90	250	120 h 50 mA cm^−2^ FE_CO_ > 80%, *E*_cell_ stays at 4.3 V	—	MEA configuration	Flow cell, zero-gap	60
CoPc2	CO from CO_2_	96	165	10.5 h −0.676 V *vs.* RHE pH 7.3 FE_CO_ 91% *j* = 20 mA cm^−2^ retained	XPS, XANES, EXAFS	1 M KOH pH 7.3, 0.5 M NaHCO_3_ pH 4, 0.5 M KCl	Flow cell	61
EtO_8_–CoPc	CO from CO_2_	95	300	24 h 150 mA cm^−2^ FE_CO_ > 90%, ΔFE_CO_ < 0.1% per h, *E*_cell_ stays at 2.6 V	Raman	Cat: 1 M KHCO_3_ An: 1 M KOH	Flow cell	45
Fe porphyrin	CO from CO_2_	>98	152	24 h 27 mA cm^−2^ (1 M KOH) FE_CO_ stays > 99% V stays at −0.16 V *vs.* RHE	EDX, XANES	1 M KOH 0.5 M NaHCO_3_	Flow cell	62
NiPc-OMe on CNTs	CO from CO_2_	99.5	300	40 h 150 mA cm^−2^ FE_CO_ stays > 99.5% *E*_cathode_ stays at −0.61 V *vs.* RHE	—	1.0 M KHCO_3_	Flow cell	46
NiPc-OMe on CNTs	CO from CO_2_	98	400	12 h 100 mA cm^−2^ FE_CO_ stays > 99% *E*_cathode_ stays at −1.26 V *vs.* RHE	—	0.5 M K_2_SO_4_ adjusted to pH 2 or 0.47 with H_2_SO_4_	Flow cell	63
[Ag(i)(4-OMe-BIAN)_2_]BF_4_	CO from CO_2_	51	84	1 h 50, 100, 200 mA cm^−2^*E*_cathode_ decreases by 100–200 mV	—	1 M KHCO_3_	Flow cell	73
[Ni(Cy^111^c)]^2+^	CO from CO_2_	63	16	2 h 25 mA cm^−2^ FE_CO_ decreases from 70 to 20%	XPS, EDX	MEA configuration	Zero-gap bipolar membrane electrolyser	74
[Mn(bipy)(CO)_3_Br]/MWCNT	CO from CO_2_	70	35	5 h −0.98 V *vs.* RHE FE_CO_ decreases from 60% to 20% *J*_CO_ decreases from 25 to 5 mA cm^−2^	SEM, EDX, FTIR, XPS, UV-vis	MEA configuration	Zero-gap electrolyser	75
Graphene-supported Cu(salan)_2_	CO and C_2_H_4_ from CO_2_	CO: 43C_2_H_4_: 27	CO: ∼25 mA C_2_H_4_: ∼10 mA	1 h −0.8, −1.1 V *vs.* RHE current stable, FEs vary 10–30%	XPS, XRD, TEM, Cu_2_O seen when graphene not used as support	1 M KOH	Flow cell	76
Co porphyin–mercurated graphyne	CO from CO_2_	100	1200	360 h −1.26 V *vs.* RHE current stays at 420 mA cm^−2^, FE decrease of 0.01%/h	IR, XPS, TEM, EXAFS, AFM, XRD, ICP	1 M KOH	Flow cell	86
[Co(tpy-O-*R*_F_)_2_]^2+^ with F-CNTs	CH_4_ from CO_2_	80	10.69	6 h FE: ∼95%, consistent, current varies by 20%	XPS, XRD, ICP, Raman, IR, DLS, UV-vis	0.5 M KHCO_3_	Flow cell	78
Fe-TPP on Ni	EtOH from CO_2_	68	21	62 h −0.3 V *vs.* RHE FE decrease by 7%, 5% decrease in immobilized Ni	SEM, SEX, XRD, XPS	0.5 M KHCO_3_	Flow cell	111
Co-porphyrin covalent organic framework, conjugated on graphene	CO from CO_2_	99	191	30 h H-cell only −0.68 V *vs.* RHE FE_CO_ stable between 90–95% *j*_CO_ decreased by 10%	TEM, EDX, Raman	1 M KHCO_3_	Flow cell	85
Calgary framework 20 (CALF20)	CO from CO_2_	94.5	32.8	20 min −0.97 V *vs.* RHE FE decreases by 20%, *j* decreases from 35 to 30 mA cm^−2^	Raman, XANES	1 M KOH	Flow cell	87
Cu-cluster based MOF (NNU-50)	CH_4_ from CO_2_	66.4	389	4 h −1.0 V *vs.* RHE *J* decrease by 10%, FE_CH4_ stable at 64–70%	XRD, Raman, SEM	1 M KOH	Flow cell	94
Cu-DBC MOF	CH_4_ from CO_2_	80	203	2.5 h −0.9 V *vs.* RHE FE_CH4_ stable at 80%, *j* stable at 200 mA cm^−2^	XRD, Raman, XPS	1 M KOH	Flow cell	95
Copper–perylene tetracarboxylic di(propyl imidazole) MOF	CO from CO_2_	70	345	71 h 80 mA cm^−2^ FE_CO_ stays between 70–75% *E*_Cell_ stays at 4.2 V	XPS, XRD, TEM	1 M KOH	Flow cell	96
Cu coordination polymer with benzimidazole units	Acetate from CO	61	400	190 h 250 mA cm^−2^ FE_acetate_ stays between 50–55%, *E*_cell_ stays at 2.8 V	XANES, IR, SEM, EDS, XPS	1 M KOH	Flow cell MEA with AEM MEA with CEM	53
Cu-TABQ polymer	C_2_H_4_ from CO_2_	63.2	423	20 h −1.07 V *vs.* RHE *J* constant at 410 mA cm^−2^, FE_C2_ decreases from 70 to 0%	SEM, TEM, XRD Cu particles form after 10 h	1 M KOH	Flow cell	97

## Reactor configurations

Prior to diving into individual systems, we provide an overview of several commonly used reactor configurations employing GDEs, and such configurations will primarily be the ones used in the literature examples highlighted later.^36,37^ This is not an all-encompassing list and promising results have been obtained with systems using configurations like porous solid electrolytes,^38^ multi-reactor configurations,^39^ or electrolysis of CO_2_ capture solutions^40^ but these are outside the scope of this current review.

### Flow cells

**Fig. 3:**
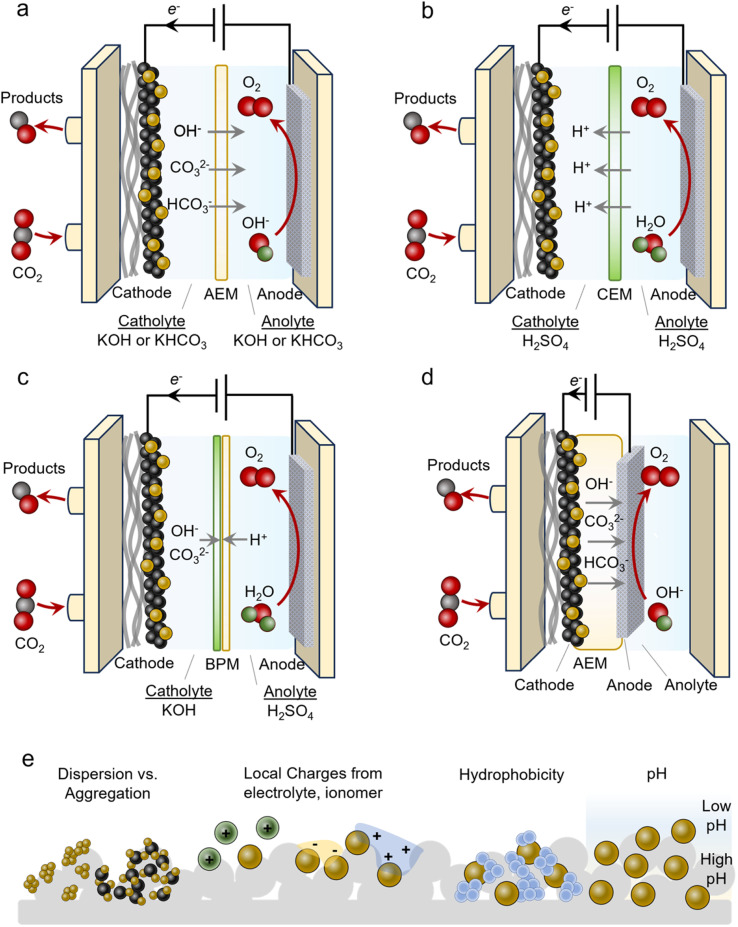
AEM-based flow cells (a) operate with alkaline or neutral electrolytes; however, carbonate formation and crossover is a challenge. Acidic CEM-based cells (b) mitigate this but suppressing the HER is a hurdle. BPM-based cells (c) may also suppress CO_2_ losses, though the membrane stability and added cell resistance are a tradeoff. MEAs (d) often feature low cell resistance but have disadvantages of carbonate/product crossover and stability. Microenvironments around the catalysts affect the system performance, as factors like dispersion, local charges, hydrophobicity, and pH modulate the reactivity (e).

An often-used geometry is that of a microfluidic flow cell. Here, a GDE is used with the reactant stream on one side and a flowing liquid catholyte on the other. The cathode chamber is separated from the anode chamber by an alkaline exchange membrane (AEM). A typical configuration employs alkaline electrolytes like 1 M KOH to minimize the HER and promote the CO_2_R (Fig. 3a). Anodes used in alkaline media are often cost-effective Ni, Fe and Co-based materials. In isolation, this is advantageous and many CO_2_R systems have reported impressive performance metrics when focusing on the cathode current and FE only, however, several technical challenges hinder their practicality. Under high operating currents (>100 mA cm^−2^), the internal resistance of the cell, which includes the anolyte, catholyte and membrane, begins to confer a significant energy penalty. Consequently, the overall operating cell voltage (*E*_Cell_) is often more than 4 V in this regime, thereby minimizing the energy efficiency (EE). An ideal system would operate at around 50% EE (∼2.4 V). The second challenge is that of CO_2_ losses due to carbonate formation.^34^ CO_2_ reacts spontaneously with OH^−^ in alkaline and neutral electrolytes to form HCO_3_^−^ and CO_3_^2−^ species. This places upper limits on how much CO_2_ can be converted into the desired products. Further, these species can cross over to the anode side, acidify the anolyte and decrease its performance and stability. Regenerating the electrolyte and recovering the CO_2_ imposes significant energy penalties and makes the overall process impractical.^36^ Because of these issues, AEM-based flow cells are more model systems rather than a technologically viable solution.

Several issues above can be circumvented by using acidic electrolytes employing cation exchange membranes (CEMs) (Fig. 3b). Such configurations do not form carbonates to the same extent because there is little OH^−^ in the bulk (even though local pH may increase under operating conditions). Membranes used here are also selectively permeable to protons, further hindering carbonates from crossing over to the anode. A hurdle to overcome is attaining selectivity for the CO_2_R in the presence of high bulk H^+^ concentrations. This can be accomplished by augmenting the near-surface cation concentration and/or depleting the H^+^ through sufficient rates of proton-consuming reactions like CO_2_R.^41^ Further, anodes used in acidic media are almost exclusively precious-metal-based (Ir and Ru), thus adding additional cost to the electrolyser. Alternatively, bipolar membranes (BPMs) can be used where CO_3_^2−^ reverts to CO_2_ at the anion exchange membrane (AEM)–cation exchange membrane (CEM) interface (Fig. 3c).^29^ Here, a neutral or alkaline environment can be maintained at the cathode with the AEM facing it (forward-bias mode). A drawback of these systems is their lack of long-term stability and additional resistance that increases *E*_Cell_. In configurations that place the CEM on the cathode side (reverse-bias mode), H^+^ accumulation can acidify the reaction environment and must also be adequately accounted for to minimize the HER.

### MEAs

An alternative to the flow cell is the MEA (Fig. 3d). In this geometry, the catalyst/GDE is pressed directly into the membrane that separates it from the anode compartment. The membrane acts as both the electrolyte and separator and here an AEM, CEM or BPM may all be used. This configuration often comes with a much lower *E*_Cell_ as catholyte and anolyte resistive pathways are eliminated. Oftentimes, *E*_Cell_ values are much lower than those of flow cells as a result, though this is not always the case. A principal challenge with these systems is the precipitation of cation (*e.g.* K^+^) or CO_3_^2−^ salts throughout the course of the operation that eventually block access to the CO_2_ reactant.^42^ Even in the absence of an aqueous catholyte, the reduction of CO_2_ to CO, for example, produces 2 OH^−^ ions that can react with CO_2_ to form carbonate species. With AEMs or BPMs with the AEM on the cathode side (forward-bias mode), the use of OH^−^ instead of CO_3_^2−^ as the anionic charge carrier is ideal for minimizing carbonate crossover that may acidify the anode and release CO_2_ in an undesired compartment. However, this is often difficult to carry out. Anionic reactants like HCOO^−^ or even neutral ones like ethanol may also be transported across the AEM. Water management is also an important factor as water in a humidified CO_2_ stream is a source of protons but an excess of it can lead to cathode flooding.^43^ Finally, membrane degradation from mechanical stress from high OH^−^ concentrations is a common aspect limiting the long-term stability of MEA systems. Thus, fully understanding the chemistry and managing and various water and ion transport mechanisms are keys to attaining highly efficient and stable systems.^44^

### Local environments

Catalyst microenvironments, as well as electrode and reactor geometry play determining roles in the systems' overall performance (Fig. 3e). Especially within GDEs that operate under high current density, the microenvironment around the catalyst can vary significantly from the bulk and also system-to-system. Because of this, the metrics given later in the review are an aggregate function of the inherent properties of catalysts, the local environment and the reactor, which render side-by-side direct comparisons of catalysts difficult.

The most common route for catalyst integration into GDEs is the drop-casting or spray deposition of a catalyst ink onto the surface of a GDE. Such an ink often contains the catalyst, conductive particles like carbon black or carbon nanotubes, and an ionomeric binder in a volatile solvent like ethanol. The component ratio as well as the GDE characteristics (porosity, hydrophobicity…) can also affect performance. For example, proper catalyst dispersion *via* the addition of conductive carbon supports is key to arriving at a uniformly covered electrode with well-dispersed catalysts that can effectively utilize each active site.^45,46^ Ionomer type and quantity also impact local ion concentrations and electric fields that modulate the reactivity, transport of reactants, local pH and active site accessibility.^41^ For example, anionic ionomers may enhance local CO_2_ concentrations, cationic ionomers may increase local pH by trapping OH^−^ near the surface and blocking bicarbonates, and these two can even be used together to realize the benefits of each.^47^ Cation augmenting layers have also been beneficial for promoting the CO_2_R in acid by stabilizing negatively charged intermediates.^48^

Added components to the catalyst ink like PTFE particles may also impact local hydrophobicity and reactant transport to active sites to enhance performance.^49,50^ The relative humidity in the CO_2_ stream also modulates performance since the number of protons and local environment are affected.^43^ In general, we note that CO_2_R reactions are proton-consuming and thus, operating at high current densities in neutral electrolytes often results in more alkaline local environments, even though this may be inhomogeneously distributed throughout the GDE.^51^ In contrast, the formation of carbonates in an alkaline medium leads to a locally buffered system with a lower pH than the bulk.^52^

### Catalyst selection criteria

Regarding the selection of catalysts for use in GDE-based systems, a more stringent set of requirements is in place. A plethora of established molecular CO_2_R catalytic motifs have been reported in past literature studies, including transition metal-based porphyrins, phthalocyanines, polypyridines, cyclams, corroles and more that can be translated over to GDEs. In general, such active sites, often with M–N_4_ coordination, feature moderate binding energies to CO_2_ and intermediates (*COO^−^, *COOH, *CO…) such that they can effectively adsorb the reactant and desorb the final products, in line with the Sabatier Principle. Similarly, they do not strongly adsorb H^+^, which would lead to selectivity for H_2_ instead. However, while such molecular CO_2_R catalysts have been shown to be stable as solubilized or heterogenized species in aqueous (mainly near-neutral buffered KHCO_3_) or non-aqueous electrolytes, they have mainly operated there under lower currents and less reductive potentials. In KOH or even in KHCO_3_ electrolytes, which can be alkaline near the electrode surface, catalysts are more prone to react irreversibly with OH^−^ ions present in high concentrations. If there are traces of O_2_, or even SOx/NOx in the gas feed, as in the case of systems directly using industrial flue-gas, reactions with these species can produce harmful byproducts like HO_2_^−^ that may further react with molecular species. Alternatively, catalysts in contact with CEMs that generate highly acidic environments should also be resistant to reactivation in the presence of high H^+^ concentrations as well as not tend to reduce H^+^ en route to undesirable H_2_ production.

Furthermore, under highly reducing conditions, often in excess of −1 V *vs.* RHE, the metal site may be irreversibly reduced and consequently form metal particles and thus, catalysts with this final reduction potential outside of a large operating window should be chosen.^53^ Highly reducing potentials also lead to catalyst desorption and thus, effective strategies for catalyst immobilization should be put in place. For example, commonly used strategies in H-cell systems of using carboxylate or phosphonate-based linkers immobilized on metal oxide surfaces or thiol-based linkers that undergo reductive desorption are not viable in these situations.^13^ Species like porphyrins and phthalocyanines strongly adsorb to graphitic carbon supports through pi–pi interactions, even under highly reducing and alkaline/basic conditions, rendering them a natural choice for investigation.^54–56^ Other catalysts, like those with alkyl or perfluoro functionalities, can be immobilized *via* their hydrophobic nature, and this may even play into generating a more favorable CO_2_R microenvironment.

Regarding the technoeconomic aspects of catalyst selection – the cost of cathode catalysts is rather small as compared with the total cost of the total electrolyser and here, the membrane and anode (if Ru or Ir is used) are more significant contributors.^7,57^ Further, the prices of the products are more sensitive to aspects like current density, stability and single pass conversion, which should be focused on, alongside CO_2_ and electricity prices. While the scalability of catalysts can vary greatly, from readily produced porphyrins^58^ to catalysts requiring extensive synthetic procedures, we argue that molecular catalysts in this context should still be primarily regarded as model systems from which lessons can be readily translated to systems like graphene/graphite-embedded M–N_4_ SACs or other heterogeneous materials that can be readily produced at scale.

## Porphyrins and phthalocyanines in GDEs

**Fig. 4:**
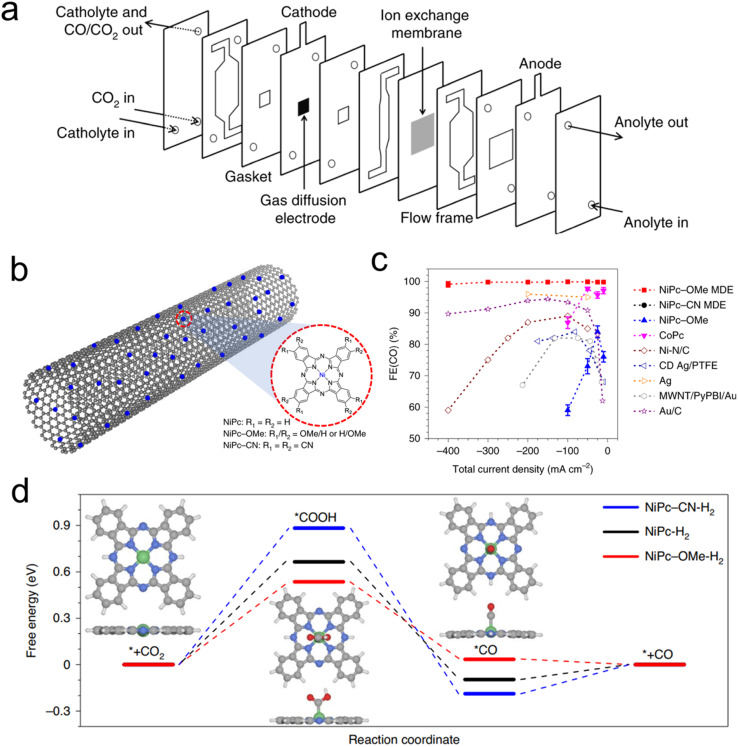
The typical electrolyser configuration employing GDEs (a),^61^ a scheme of catalyst-CNT integration (b), effects of functional groups on performance (c), and free-energy landscape of CO_2_R (d),^46^ reproduced with permission from Springer Nature copyright 2019.

Porphyrin and phthalocyanines are effective CO_2_R catalysts that lend themselves very well to heterogenization. They feature strong attraction to graphene, carbon nanotubes (CNTs) and similar carbonaceous surfaces that can act as conductive supports due to pi–pi interactions.^54–56^ Catalyst inks of the molecule/carbon mixture are then readily deposited on GDEs for use in flow cells or MEAs (Fig. 4a). Initial studies used cyano-substituted Co phthalocyanines loaded on CNTs that were then deposited on carbon paper-based GDEs in an AEM-based flow cell. The tendency of these catalytic sites to be active for CO_2_R to CO conversion could be maintained, and CO partial current densities as high as 82 mA cm^−2^ could be reached.^59^ A subsequent study used unsubstituted Co phthalocyanines, attached to a standard carbon powder, and demonstrated their viability in a zero-gap MEA employing an AEM. In this configuration, CO production at 175 mA cm^−2^ was reached, at a full cell potential of only 2.5 V.^60^ The MEA system's performance declined after 8 h, the same catalyst in a flow cell lasted 120 h at 50 mA cm^−2^ in a neutral-pH flow cell.

A follow up study demonstrated that functionalizing the Co phthalocyanine with a trimethylammonium group endowed the system with a high CO production rate over a wide (4–14) pH range in a flow cell configuration.^61^ Finally, using an octaethoxy Co phthalocyanine and maximizing catalyst dispersion improved the connectivity to each active site, as demonstrated by *operando* Raman spectroscopy.^45^ This, in turn, led to CO partial current densities of approx. 330 mA cm^−2^ in a flow cell with a KHCO_3_ catholyte while also improving the system longevity to 24 h at 150 mA cm^−2^ with minimal performance loss. This study highlighted the effects of the catalyst microenvironment and showed how the measured turnover frequency of the catalysts is largely dependent on their dispersion/aggregation on the catalyst surface, which could be improved through the addition of porous carbon particles to the catalyst ink.

Beyond Co phthalocyanines, Fe Porphyrins with trimethylammonium groups have been implemented for CO production from CO_2_ in a flow cell.^62^ This particular system required a mere 50 mV overpotential for the CO_2_R, resulting in a full cell energy efficiency of 71%, though only at a modest current density of 27 mA cm^−2^. This performance was retained for 24 h. On the other hand, attaining higher current densities (max of 152 mA cm^−2^) required 470 mV. Methoxy-functionalized Ni phthalocyanines, loaded onto CNTs were a particularly active system in terms of maximal CO production in a neutral electrolyte flow cell. The initial system exhibited CO partial current densities of almost 400 mA cm^−2^ in neutral electrolyte and featured selectivity and cathodic overpotential for 40 h at 150 mA cm^−2^.^46^ One important aspect of this work is the importance of microenvironments. The use of CNTs is crucial for catalyst dispersion while the integration of PTFE particles in the catalyst ink enhanced activity by conferring a more hydrophobic catalytic environment and augmenting CO_2_ diffusion pathways to the active sites. Beyond high catalytic performance, a particular strength of this work is the combined spectroscopic and theoretical investigation of a series of experimentally evaluated phthalocyanines with various functional groups, a study made possible by having a well-defined and modular molecular active site (Fig. 4b). Through X-ray absorbance measurements and computational modelling, the authors determined that the methoxy groups led to an electron-rich environment on the Ni active sites enhanced the Ni–N bond strength and consequently the stability of the molecule while accelerating CO desorption, and therefore CO_2_R catalysis (Fig. 4c and d). A follow-up work investigated this catalyst in a flow cell employing acidic electrolytes (as low as pH 0.47) and found that just as high CO partial current densities as before (almost 400 mA cm^−2^) could be attained with near unity selectivity, in addition to constant selectivity and overpotential for 12 h at 100 mA cm^−2^.^63^ A key here was the addition of K^+^ ions, which stabilized the CO_2_R intermediates en route to CO formation.^48,64^ This system configuration was particularly appealing as the use of acidic electrolytes minimizes carbonate formation that leads to carbon crossover to the anode, excess energy costs to regenerate the electrolyte, and low single pass conversion rates.^34,41^

Porphyrins have been implemented as key components of tandem electrocatalytic systems. The capacity for CO production of such molecules is often higher than metals like Cu and this is readily exploited. A highlight is the use of a Fe-porphyrin as a CO-generating unit, adsorbed onto a Cu catalyst that subsequently performed C–C coupling from the CO.^65^ The local enrichment of CO augmented the production rate (up to 124 mA cm^−2^) and FE (up to 41%) of ethanol in the tandem system by approx. 10–20% relative to the unfunctionalized Cu. In keeping with the concept of CO production at the first site and its further reduction at a second site, efficient CH_4_ production was also realized.^66^ In this system, a Co phthalocyanine reduced CO_2_ to CO while an adjacent Zn–N_4_ SAC performed the CO to CH_4_ conversion. In all, a FE_CH4_ of 18% at a current density of 44 mA cm^−2^ was attained. Interestingly, the Co phthalocyanine had a dual role of providing CO but also promoting the accumulation of *H on the N-sites of the Zn–N_4_, which subsequently hydrogenated *CO adsorbed on the Zn en route to CH_4_ as the final product. This study highlights the importance of not only effectively integrating the two catalytic components (*i.e.*, proximity, dispersion…) but also being cognizant of the secondary effects that they may have on each other.

### Relation to M–N_4_ single atom catalysts

While a comprehensive discussion of M–N_4_ catalysts embedded in carbon layers is outside of the scope of this work, we use this subsection to discuss their relationship to the porphyrin and phthalocyanine catalysts detailed above. In general, M–N_4_ catalysts are often (though not always) synthesized through the controlled pyrolysis of N- and C-containing molecules or MOFs, together with a transition metal source.^21–23^ This route is readily scalable and yields a catalyst powder and is thus attractive from an industrial standpoint. The active site of such systems is also an M–N_4_ site, though the surrounding carbon macrocycle may vary. Other heteroatoms like S, O, or P can also be used to replace one or more N atoms to modify the site's activity. Both transition metal-containing porphyrins/phthalocyanines and M–N_4_ SACs feature exceptional activity for CO_2_ reduction to CO, and recently, M–N_4_ SACs have been integrated into GDE-based CO_2_R reactors for selective CO production at industrially relevant current densities.^39,67,68^ One limitation to the aforementioned SACs is that the synthesis often yields a mixture of active site configurations^69^ and generally, the complexity and secondary coordination sphere of the active sites is more limited as compared to molecular catalysts.^70–72^ While purely molecular catalysts are often not as scalable and industrially translatable, we contend that they are an important model system for precise studies of structure–activity relationships to extract lessons that can subsequently be incorporated into industrially ready platforms.

## Extended classes of molecular catalysts

**Fig. 5:**
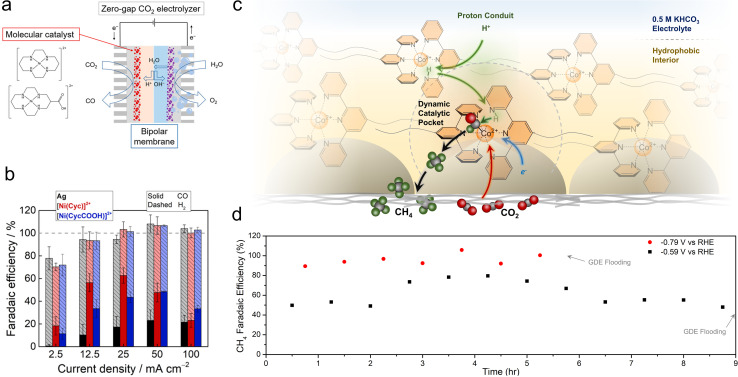
Ni cyclam catalysts were integrated with bipolar membrane electrolysers (a), in which they reduced CO_2_ to CO (b).^74^ Proton shuttling enables CO_2_R within a hydrophobic pocket within GDE electrodes (c) and shifts the reaction selectivity to CH_4_, with a selectivity of almost unity (d).^78^ Reproduced with permission from the American Chemical Society, copyright 2022.

While porphyrins and phthalocyanines are the most popular classes of catalysts thus far, other systems have also exhibited intriguing reactivity. Ag-based complexes, similar to Ag metal, were found to be selective for CO production in a flow cell employing neutral electrolytes, with partial current densities of up to 84 mA cm^−2^, though only 1 h of continuous catalysis was shown.^73^ Ni cyclam catalysts were integrated with bipolar membranes in a zero-gap MEA configuration for the CO_2_R to CO (Fig. 5a).^74^ A reverse bias configuration was used that featured the CEM on the cathode side and the AEM on the anode side. The selectivities (20–60%) and CO partial current densities (approx. 35 mA cm^−2^) were modest (Fig. 5b), which enabled the use of humidified CO_2_ and water as the only feedstocks and eliminated CO_2_ losses to carbonate formation. The performance in this configuration was impressive because the CEM rendered the local environment acidic. A challenge here was that the system gradually lost its selectivity for CO within 2 h. An improvement was made with a subsequent generation of Mn carbonyl complexes in a similar setup, in which CO was produced with 70% selectivity and partial current densities of 35 mA cm^−2^.^75^ However, the FE for CO decreased from 60% to 20% within 5 h.

Cu-salan complexes, investigated in an alkaline flow cell, on the other hand, produced CO at low overpotentials (maximum of approx. 45 mA cm^−2^) but began producing multi-carbon products such as ethylene, ethanol, and acetate at potentials more negative than −0.7 V *vs.* RHE.^76^ This particular observation is mechanistically very interesting since C–C coupling on single-site catalytic systems is rare and usually proceeds through *CH_*x*_O–CH_*x*_O coupling on adjacent sites instead. Post-catalysis characterization showed that Cu_2_O was detected in the absence of a graphene support, which likely stemmed from the oxidation of Cu particles that were formed during catalysis. However, the graphene-supported complexes did not show evidence of aggregation through XPS, XRD or TEM analysis, though only 1 h of performance stability was shown. Nonetheless, the observation of C_2_ products brings into question whether the complexes could have reversibly formed aggregates then re-dispersed into isolated units, a phenomenon observed with Cu SACs embedded in carbon supports.^77^

Co terpyridine catalysts functionalized with perfluoroalkyl side chains also exhibited unique reactivity at the gas–liquid–solid interface found within GDEs.^78^ While this catalyst produced CO as a solubilized homogeneous species in organic electrolytes, its selectivity shifted towards CH_4_ when immobilized on GDE surfaces. Analytical and spectroscopic experiments revealed that the ordered nature brought about by the perfluoro–perfluoro interactions led to a proton shuttling mechanism to hydrogenate the CO_2_R intermediates within a hydrophobic catalytic pocket (Fig. 5c). The optimized system, when integrated with fluorine functionalized CNTs, was able to produce CH_4_ with nearly 100% selectivity and partial current densities of approx. 10 mA cm^−2^ in a flow cell, with stability upwards of 5 h, until electrode flooding prevented longer stability runs (Fig. 5d).

While there are not many studies beyond CO_2_R within the context of molecular GDE systems, one exceptional study focused on H_2_ oxidation in an acidic flow cell, a fuel-cell-relevant reaction.^79^ The authors used CNTs with pyrene butyric acid surface functionalization. This enabled them to load a Ni-based molecular catalyst that was attracted to the modified surfaces through electrostatic interactions and deposit the film on a gas diffusion electrode. With this system, the HOR current density of approx. 400 mA cm^−2^ was attained. A translatable aspect of this work is the use of tailored electrostatic interactions to assemble molecular catalytic layers, particularly relevant to systems that cannot take advantage of pi–pi interactions common to porphyrins and phthalocyanines.

## Molecular catalyst containing extended systems

Beyond isolated molecules, molecular sites embedded within a MOF/COF/polymer scaffold are an interesting platform to study as they offer control over the chemistry of the active site and 1st coordination sphere common to molecular catalysts while control over the pore/linker chemistry can further modulate reactivity through the secondary coordination sphere and larger environmental effects. In purely aqueous CO_2_R systems, examples of this include the use of pyridinium^80^ and trimethylammonium^81^ motifs to stabilize *CO_2_ and pyridine units to function as proton relays.^82^ While out of the scope of this particular perspective, MOFs may also have utility in modulating the coordination environment and local transport properties around a secondary catalyst.^83,84^

Incorporating extended structures in GDEs can be accomplished by similarly taking advantage of pi–pi interactions, as demonstrated through two-dimensional porphyrin sheets loaded onto graphene in a neutral electrolyte flow cell.^85^ In this particular example, electronic synergy between the catalyst layer and substrate, namely the electron-withdrawing effects from the graphene that rendered the Co porphyrin active sites more electron deficient, accelerated the CO_2_R catalytic process to generate CO with partial current densities of approx. 191 mA cm^−2^. This is a highlight of how both the catalyst and its support play crucial roles in the system's activity and stability. In this same direction, mercurated Co-porphyrin graphyne sheets synergistically gave rise to a high-performing CO_2_ to CO production system in a flow cell (FE_CO_ of 100% at 1.2 A cm^−2^).^86^ The mercurated graphyne was determined to play a key function in suppressing the HER and promoting *COOH formation by modulating the electronic structure of the Co porphyrin's active site. Importantly, this is also one of the few studies that performed stability measurements at both currents of approx. 420 and 1000 mA cm^−2^, with the former being for 360 h with very minimal performance decay, more closely mimicking practical conditions; an extensive list of post-catalysis characterizations was also performed to ensure that the system retained its molecular nature.

**Fig. 6:**
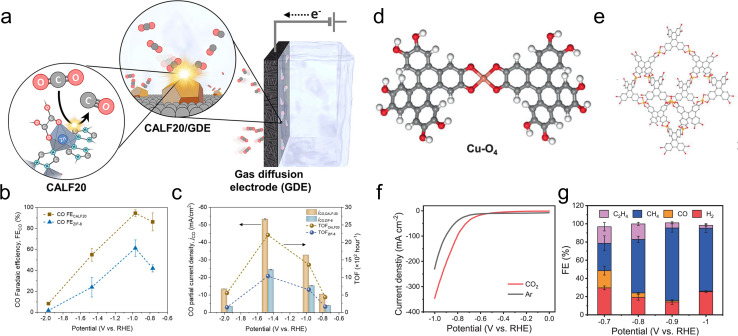
Zn-based CALF20 features ligand-based active sites (a) whose electron-rich structure facilitates CO_2_R to CO more than a ZIF-8 analogue (b and c).^87^ Cu-O_4_ active sites (d) within a Cu-BTC MOF (e) exhibit both high activity (f) and selectivity (g) for CO_2_R to CH_4_.^95^ Reproduced with permission from the American Chemical Society, copyright 2021 and Springer Nature, copyright 2021.

Another study used a MOF, CALF20, with Zn nodes and azole ligands as the CO_2_R active sites in an alkaline flow cell (Fig. 6a).^87^ On comparison to a standard structure comprised of Zn nodes and imidazole linkers, ZIF-8, the superior performance of CALF20 for CO_2_R (Fig. 6b and c) was attributed to the more electron-rich triazole ligands (*vs.* diazole for ZIF-8) that promoted the formation of the *COOH intermediate en route to CO production. Only 20 minutes of stability measurements were shown and the integrity of the MOF for longer durations remains an open question. The effect of linkers was also used to modulate the reactivity of Ni-based 2D sheets used for oxygen reduction to peroxide, a widely used chemical oxidant.^88^ A hypothesis was that amine-functionalized linkers weakened the binding of *OOH on the Ni active sites and thereby promoted the desorption of the desired product (partial current densities of up to 200 mA cm^−2^) instead of over-reduction to H_2_O in a GDE alkaline flow cell setup.

Cu is an often-used element for CO_2_R because it can produce products beyond CO.^89^ Molecular Cu active sites also lead to a wide variety of CO_2_R products as a function of their particular coordination and reaction environments. However, great care must be taken to understand the actual active structure of the catalyst as Cu-based materials, in particular, tend to restructure under reductive conditions,^90–93^ and, as detailed later, one must adequately characterize the system to claim the retention of the molecular nature of active sites. One interesting example entails Cu hexanuclear cluster active sites within a NNU-50 MOF that produced CH_4_ with current densities as high as 400 mA cm^−2^ in an alkaline flow cell.^94^ This performance was attributed to the strong interactions between the Cu(i) sites and CO_2_ reactant. While the performance was stable over 4 h and Raman and XRD analysis revealed the retention, at least in part, of the organic linkers and crystallinity, it would still be worthwhile to probe any dynamic nature of the Cu clusters and whether there is full reduction to Cu(0) and small particle formation over longer periods, and how much of the Cu is still in the as-synthesized coordination presumed to be the active site.

CH_4_ was also the principal product for a Cu-DBC MOF with Cu-O_4_ active sites tested in an alkaline flow cell (Fig. 6d and e).^95^ CH_4_ partial current densities as high as 203 mA cm^−2^ and high selectivity were observed (Fig. 6f and g) and attributed to a thermodynamically favorable CO_2_R pathway on the particular Cu-O_4_ catalytic motif. This was experimentally verified since Cu-O_4_ sites within a Cu-HHTP MOF showed similar selectivity while Cu-N_4_ sites within porphyrin and phthalocyanine-based COFs were not as selective. Although only 3 h of stability were demonstrated under high currents (∼200 mA cm^−2^), XRD, Raman and XPS measurements indicated a retention of the MOF structure. For both examples, stability under longer operational times (100 s or h) would be ideally measured since practical systems would have to operate for tens of thousands of hours.

A series of Cu-active sites within perylene tetracarboxylic di(propyl imidazole) (PDI) structures were evaluated in an alkaline flow cell for CO_2_R with particular attention paid to their coordination environment.^96^ Cu–N coordinated sites were selective for CO, Cu–C sites selective for CH_4_ while Cu–Cu sites in larger clusters produced a mixture of CH_4_ and C_2_H_4_. Theoretical modelling suggested that Cu–N sites desorbed *CO readily, leading to high CO production while Cu–C sites promoted further hydrogenation steps en route to CH_4_, and Cu–Cu sites enabled the adsorption of more than one intermediate for C–C coupling en route to C_2_H_4_ production.

Cu(i) sites within a benzimidazole coordination polymer were applied in CO reduction (COR).^53^ Acetate could be produced with partial current densities as high as 240 mA cm^−2^. Interestingly, the isolated Cu sites could accommodate the adsorption of 2 CO molecules and facilitate C–C coupling to produce acetate as the final product. The performance of the system in terms of selectivity and cell voltage was shown to be stable for 190 h at 150 mA cm^−2^. There are several notable strong points to this work. First, the stability of the catalyst was evaluated with both *ex situ* (Raman, XAS, IR) and *in situ* (Raman, XAS) methods that precluded the formation of Cu particles. Electrochemical and DFT analyses were combined to show that the reduction of the Cu(i) sites did not occur until there were very negative potentials outside of the operating window of the catalytic measurements. Finally, the catalyst was integrated into a more technologically practical CEM-based MEA that prevented the crossover of the acetate ion while also enhancing the system's longevity by preventing flooding of the cathode.

A Cu(ii) single site-based coordination polymer was found to be active for CO_2_R to C_2_H_4_.^97^ A key reason for this performance was found to be the tetraminobenzoquinone (TABQ) linkers broadening the D-band of the Cu and, thereby, promoting the binding of *CO and its subsequent C–C coupling en route to the final C_2_H_4_ product with rates of up to 423 mA cm^−2^. In this example, Cu particles were evidenced after 10 h of measurement, though not necessarily before. However, a key question remains as to whether the initial 10 h still entailed a partial transformation of the initial structure into a small Cu cluster formation that could not yet be detected with measurements like XRD.

## Future directions with molecular systems

### Molecular tuning of primary and secondary coordination spheres

**Fig. 7:**
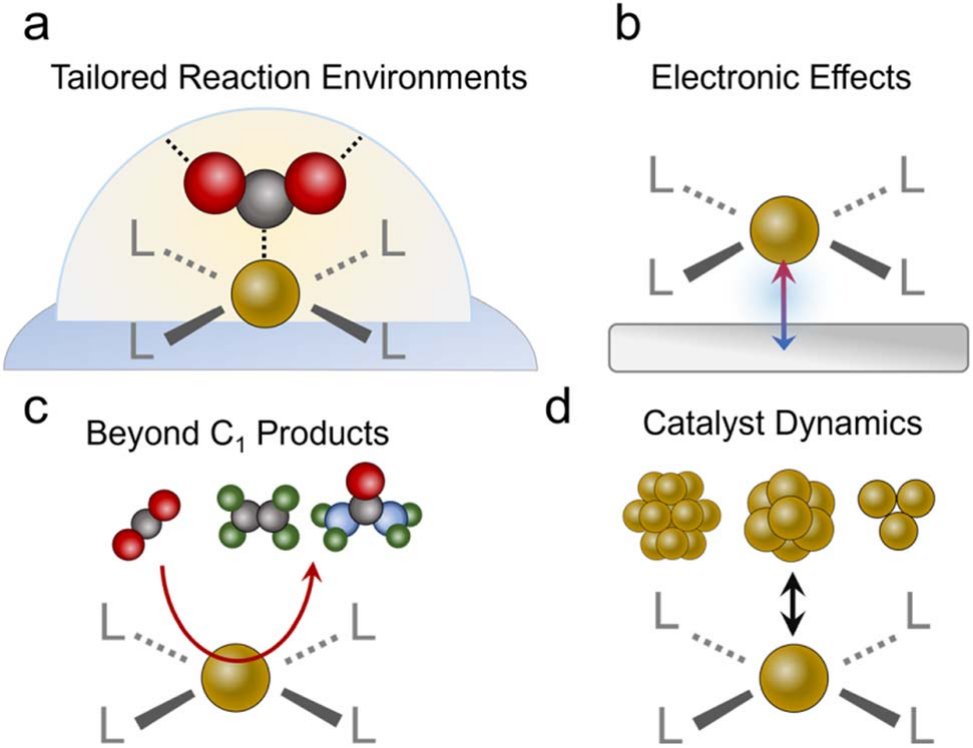
Strategies to be pursued entail designing both active sites as well as coordination environments (a), harnessing electronic effects from conductive supports (b), developing strategies to carry out C–C and C–N/S bonds (c), and a thorough evaluation of possible catalyst decomposition and actual catalytic motifs contributing to the observed activity (d).

After concluding the overview of the progress molecular-based systems have made to date, we will identify exciting new avenues to pursue. We begin with a re-emphasis on the unique strengths conferred by molecular catalysts in not only having well-defined active sites but being able to rationally tune the coordinating ligands and the reaction environment (Fig. 7a). While several studies have made inroads in taking advantage of these attributes, such as the study on substituted Co^61^ and Ni^46^ phthalocyanines and Cu complexes,^96^ this area remains largely unexplored relative to work done with molecules in more conventional reaction setups. There is a particular opportunity with catalysts like MOFs to explore reaction environments such as pore chemistry, size, and more, which can be orthogonally tuned to a greater extent than pure molecules. Important questions to answer would be whether improvements in catalytic activity through modulation of the 1st and 2nd coordination sphere in aqueous systems translate to the gas–liquid–solid interfaces in GDEs, and how? In these environments, do the same considerations apply to tuning hydrophobicity and hydrogen bonding/electrostatic stabilization of intermediates? Do these insights parallel those found in studies that functionalized the surfaces of heterogeneous electrocatalysts,^98,99^ and if not, what are the fundamental origins of the discrepancies? Further, can the unique selectivity of molecular sites be harnessed for CO_2_R in low CO_2_ concentrations^100^ or in the presence of impurities?^101^ For example, the presence of O_2_,^102^ SO_*x*_,^103,104^ and NO_*x*_^105^ may have detrimental effects on the current density, selectivity and/or stability of heterogeneous catalysts.^106^ The discovery of impurity-tolerant molecular sites provides insights that can be translated to analogous heterogeneous systems, and initial work with O_2_-tolerant cobalt phthalocyanines is a promising step in this direction.^107,108^

Beyond tuning the active sites themselves, we encourage the community to explore avenues for synergy between the molecules and their supports (Fig. 7b). Work with molecular systems in conventional electrochemical cells showed how, in select cases with good electronic communication, the discrete electronic states of the molecule disappear and instead the system adopts the extended electronic structure of the conductive substrate.^109,110^ This may already be the case for some porphyrins and phthalocyanines loaded on CNTs, graphene and their analogues. A very recent study showed how ethanol can be produced from CO_2_R *via* a Fe porphyrin adsorbed on a Ni surface.^111^ Here, the Ni substrate fixed the Fe oxidation to the Fe(ii) state throughout the catalytic cycle, which led to strong Fe–CO binding and consequently, enabled the further reduction of this species to ethanol as the end product. Generally, the development of rational strategies to connect molecular catalysts to an electrode and promote (or minimize) such effects would be an impactful addition to the field.

### Beyond C_1_ chemistry

While most CO_2_R reactions with the systems above produce CO, with a few notable exceptions,^76,78,97,111^ an upcoming challenge would be to develop additional strategies for C_2_ (*e.g.*, ethylene or ethanol) or more complex product generation (Fig. 7c). To this end, C–C bonding will have to be carried out on a single catalytic site, which can often only accommodate one intermediate while minimizing the desorption of C_1_ intermediates, another typical characteristic of molecular active sites. This is in contrast to heterogeneous catalysts like Cu, which can feature a dense surface coverage of pre-coupled intermediates like *CO. An opportunity exists here for the use of molecular catalysts in tandem with catalytic schemes. Since CO is readily generated with high selectivity on porphyrins, phthalocyanines and similar systems, they have been effectively coupled with Cu catalysts to augment the systems' production of C_2_ products.^39,65^ This concept can be readily extended to include molecular CO reduction catalysts^53^ as the 2nd site to maximize the selectivity for a single C_2_ product. There is, however, much work to be done in developing more effective molecular CO reduction catalysts since there are not many studies doing this with GDE-based systems. Photochemical and electrochemical studies have previously shown that methane and methanol can be produced from CO.^112–114^ Success here would further enable molecular CO reduction catalysts to be combined with technologies like high-temperature electrolysis of CO_2_ to CO, which also do not have issues of carbonate formation. Beyond this, molecular active sites can be used for CO_2_ fixation beyond the simple CO_2_R such as the generation of products with C–N bonds (*e.g.* urea, amines, amides…)^115^ or C–S bonds (sulfides, sulfonates…).^116^ To this end, a molecular site can be designed to partially reduce CO_2_ to an electrophilic CO_2_R intermediate (*e.g.* CHO) that can then undergo nucleophilic attack by a solution or gas phase nucleophile (*e.g.* NH_3_, SO_3_^2−^, NH_2_OH…).

### System stability

We stress that claims of activity (particularly C_2_ products or more) must be accompanied by a rigorous evaluation of the catalyst's structure during and after catalysis. This can best be accomplished through a combination of complementary analytical techniques to understand the catalytic motif (molecular sites, clusters, particles…) that is responsible for the measured activity.^77,117^ Molecular catalysts, organic frameworks, and heterogeneous catalysts can all undergo various structural/chemical changes under reaction conditions, where capturing the ‘real’ catalytic state of the system is key (Fig. 7d).^118,119^ We also note that incomplete decomposition may also lead to a fraction of restructured catalyst that contributes the majority of catalysis while post-catalysis characterization would still detect signatures of the residual original species, thereby pressing the need for a comprehensive, multi-modal evaluation. For example, if a part of the catalyst is converted to amorphous metal clusters, techniques like infrared or Raman spectroscopy may still detect vibrational modes from the residual original species and X-ray diffraction may not detect new crystalline phases, but X-ray photoelectron spectroscopy may pick up new electronic states of the particular transition metal and elemental analysis techniques could also detect leached ions in the electrolyte solution. To this end, we encourage future studies to use complementary characterization techniques post-catalysis that include XPS/XANES to probe the electronic structure, XRD to investigate the possible formation of nanoparticles, and vibrational spectroscopy to record any changes in the organic ligands. Catalyst retention/leaching should also be quantified through ICP analysis of the electrolyte and electrochemical evaluations such as a comparison of catalyst redox peaks before and after catalytic runs. Furthermore, as some systems, especially Cu-based ones,^77^ undergo reversible restructuring from single sites to clusters, *operando* characterization should be added whenever possible as definitive proof of the retention of the molecular/single site character of the material. Here, a final system that also integrated Cu particles for ethylene production remained stable for 250 h at high currents (200 mA cm^−2^ for acetate + ethylene), whereas the flow cell configuration flooded and primarily generated H_2_ within 2 h of operation.

We note that few catalysts have attained stability of over 100 h at 300 mA cm^−2^ or more. We encourage the community to test stability under these more realistic and demanding conditions, as opposed to lower voltages/currents that naturally give more favorable results; mechanisms for system failure should be investigated as well. Sometimes the limit of stability is a result of catalyst degradation under highly reductive conditions.^62^ When observed, the possible routes/mechanisms of degradation should be discussed as this would be valuable knowledge to add to the community. At other times, it is the electrolyser itself that fails. For example, a CoPc catalyst containing a MEA cell diminished due to carbonate salt precipitation at 10 h but the same catalyst maintained the majority of its performance for 120 h in a flow cell.^60^ Identifying the root cause and mechanism would encourage more work on how to overcome these challenges.

### The role of molecular catalysts in next-generation CO_2_R

Looking ahead, we discuss what the role of molecular catalysts may be. As mentioned above, molecular catalysts can be tuned to an unparalleled degree and thus are excellent models for investigating structure–activity relationships in various environments found in GDE-based systems. An important point here is that such environments can be significantly different than what is found in H-cells that operate under lower currents and lack a direct interface with the reactant gas stream. Such insights can then be translated over to industrially translatable platforms like M–N_4_ SACs. As this work has shown, molecular catalysts have been integrated into a variety of different reactors. While the focus has been on flow cells, there are several studies on molecular catalysts in the more industrially ready MEAs and thus, we encourage further exploration in this area since there are still many challenges in this direction to be overcome in the wider CO_2_R field. There are only a few examples of molecular systems operating in acidic electrolytes or interfaced with CEMs in an acidic environment. Here is where the tunability and selectivity of molecular catalysts over the HER can be fully leveraged. Success with these systems that are not limited by carbonate formation can help pave the way, for example, to practical, industrially translatable acid/CEM-based CO_2_R technologies that are still challenging to accomplish with heterogeneous catalysts.

## Concluding remarks

This perspective highlights an emerging area in molecular catalyst-based GDEs, particularly, the advent of functional systems and new challenges to undertake. Principal contributions here have all emerged in 2019 or later and thus, there is plenty of room to grow through fully exploiting the unique advantages conferred by molecular catalysts, both in maturing such systems towards improved performance on par with state-of-the-art heterogeneous analogues and in generating key insights that can be translated to a multitude of systems of interest to the wider community. As outlined above, there is a myriad of exciting opportunities to explore to fully harness the inherent advantages of molecular catalysts in GDE-based electrocatalytic systems and use the insights obtained to push the wider CO_2_R field ahead.

## Author contributions

All authors contributed to the conception of the work and writing of the manuscript.

## Conflicts of interest

The authors declare no competing interests.

## Supplementary Material

## References

[cit1] Fechete I., Wang Y., Védrine J. C. (2012). Catal. Today.

[cit2] Cornils B., Herrmann W. A. (2003). J. Catal..

[cit3] Poovan F., Chandrashekhar V. G., Natte K., Jagadeesh R. V. (2022). Catal. Sci. Technol..

[cit4] Smith C., Hill A. K., Torrente-Murciano L. (2020). Energy Environ. Sci..

[cit5] Bicer Y., Dincer I., Zamfirescu C., Vezina G., Raso F. (2016). J. Cleaner Prod..

[cit6] De Luna P., Hahn C., Higgins D., Jaffer S. A., Jaramillo T. F., Sargent E. H. (2019). Science.

[cit7] Shin H., Hansen K. U., Jiao F. (2021). Nat Sustainability.

[cit8] Wang M., Khan M. A., Mohsin I., Wicks J., Ip A. H., Sumon K. Z., Dinh C.-T., Sargent E. H., Gates I. D., Kibria M. G. (2021). Energy Environ. Sci..

[cit9] Ross M. B., De Luna P., Li Y., Dinh C.-T., Kim D., Yang P., Sargent E. H. (2019). Nat. Catal..

[cit10] Vogt C., Weckhuysen B. M. (2022). Nat. Rev. Chem.

[cit11] Mitchell S., Qin R., Zheng N., Pérez-Ramírez J. (2021). Nat. Nanotechnol..

[cit12] Costentin C., Robert M., Savéant J.-M. (2013). Chem. Soc. Rev..

[cit13] Dalle K. E., Warnan J., Leung J. J., Reuillard B., Karmel I. S., Reisner E. (2019). Chem. Rev..

[cit14] Nichols A. W., Machan C. W. (2019). Front. Chem..

[cit15] Leung J. J., Warnan J., Ly K. H., Heidary N., Nam D. H., Kuehnel M. F., Reisner E. (2019). Nat. Catal..

[cit16] Costentin C., Drouet S., Robert M., Savéant J.-M. (2012). Science.

[cit17] Furukawa H., Cordova K. E., O'Keeffe M., Yaghi O. M. (2013). Science.

[cit18] Leung J. J., Vigil J. A., Warnan J., Edwardes Moore E., Reisner E. (2019). Angew. Chem., Int. Ed..

[cit19] Kuruvinashetti K., Li J., Zhang Y., Bemana H., McKee M., Kornienko N. (2022). Chem. Phys. Rev..

[cit20] Heidary N., Harris T. G. A. A., Ly K. H., Kornienko N. (2019). Physiol. Plant..

[cit21] Wang A., Li J., Zhang T. (2018). Nat. Rev. Chem.

[cit22] Nguyen T. N., Salehi M., Le Q. V., Seifitokaldani A., Dinh C. T. (2020). ACS Catal..

[cit23] Chen Y., Ji S., Chen C., Peng Q., Wang D., Li Y. (2018). Joule.

[cit24] Costentin C., Nocera D. G., Brodsky C. N. (2017). Proc. Natl. Acad. Sci. U.S.A..

[cit25] Corbin N., Zeng J., Williams K., Manthiram K. (2019). Nano Res..

[cit26] Kim M., Yi J., Park S.-H., Park S. S. (2023). Adv. Mater..

[cit27] Peng Y., Sanati S., Morsali A., García H. (2023). Angew. Chem., Int. Ed..

[cit28] Zhang H., Nai J., Yu L., Lou X. W. (2017). Joule.

[cit29] Lees E. W., Mowbray B. A. W., Parlane F. G. L., Berlinguette C. P. (2022). Nat. Rev. Mater..

[cit30] Nguyen T. N., Dinh C.-T. (2020). Chem. Soc. Rev..

[cit31] García de Arquer F. P., Dinh C.-T., Ozden A., Wicks J., McCallum C., Kirmani A. R., Nam D.-H., Gabardo C., Seifitokaldani A., Wang X., Li Y. C., Li F., Edwards J., Richter L. J., Thorpe S. J., Sinton D., Sargent E. H. (2020). Science.

[cit32] Masel R. I., Liu Z., Yang H., Kaczur J. J., Carrillo D., Ren S., Salvatore D., Berlinguette C. P. (2021). Nat. Nanotechnol..

[cit33] Mekhilef S., Saidur R., Safari A. (2012). Renewable Sustainable Energy Rev..

[cit34] Rabinowitz J. A., Kanan M. W. (2020). Nat. Commun..

[cit35] Torbensen K., Joulié D., Ren S., Wang M., Salvatore D., Berlinguette C. P., Robert M. (2020). ACS Energy Lett..

[cit36] Ozden A., García de Arquer F. P., Huang J. E., Wicks J., Sisler J., Miao R. K., O'Brien C. P., Lee G., Wang X., Ip A. H., Sargent E. H., Sinton D. (2022). Nat Sustainability.

[cit37] Wakerley D., Lamaison S., Wicks J., Clemens A., Feaster J., Corral D., Jaffer S. A., Sarkar A., Fontecave M., Duoss E. B., Baker S., Sargent E. H., Jaramillo T. F., Hahn C. (2022). Nat. Energy.

[cit38] Zhu P., Wang H. (2021). Nat. Catal..

[cit39] Möller T., Filippi M., Brückner S., Ju W., Strasser P. (2023). Nat. Commun..

[cit40] Zhang Z., Lees E. W., Habibzadeh F., Salvatore D. A., Ren S., Simpson G. L., Wheeler D. G., Liu A., Berlinguette C. P. (2022). Energy Environ. Sci..

[cit41] Li J., Kornienko N. (2022). Chem Catal..

[cit42] Sassenburg M., Kelly M., Subramanian S., Smith W. A., Burdyny T. (2023). ACS Energy Lett..

[cit43] Hoof L., Thissen N., Pellumbi K., junge Puring K., Siegmund D., Mechler A. K., Apfel U.-P. (2022). Cell Rep. Phys. Sci..

[cit44] Salvatore D. A., Gabardo C. M., Reyes A., O'Brien C. P., Holdcroft S., Pintauro P., Bahar B., Hickner M., Bae C., Sinton D., Sargent E. H., Berlinguette C. P. (2021). Nat. Energy.

[cit45] Ren S., Lees E. W., Hunt C., Jewlal A., Kim Y., Zhang Z., Mowbray B. A. W., Fink A. G., Melo L., Grant E. R., Berlinguette C. P. (2023). J. Am. Chem. Soc..

[cit46] Zhang X., Wang Y., Gu M., Wang M., Zhang Z., Pan W., Jiang Z., Zheng H., Lucero M., Wang H., Sterbinsky G. E., Ma Q., Wang Y.-G., Feng Z., Li J., Dai H., Liang Y. (2020). Nat. Energy.

[cit47] Kim C., Bui J. C., Luo X., Cooper J. K., Kusoglu A., Weber A. Z., Bell A. T. (2021). Nat. Energy.

[cit48] Huang J. E., Li F., Ozden A., Sedighian Rasouli A., García de Arquer F. P., Liu S., Zhang S., Luo M., Wang X., Lum Y., Xu Y., Bertens K., Miao R. K., Dinh C.-T., Sinton D., Sargent E. H. (2021). Science.

[cit49] Rabinowitz J. A., Ripatti D. S., Mariano R. G., Kanan M. W. (2022). ACS Energy Lett..

[cit50] Xing Z., Hu L., Ripatti D. S., Hu X., Feng X. (2021). Nat. Commun..

[cit51] Böhme A., Bui J. C., Fenwick A. Q., Bhide R., Feltenberger C. N., Welch A. J., King A. J., Bell A. T., Weber A. Z., Ardo S., Atwater H. A. (2023). Energy Environ. Sci..

[cit52] Lu X., Zhu C., Wu Z., Xuan J., Francisco J. S., Wang H. (2020). J. Am. Chem. Soc..

[cit53] Luo M., Ozden A., Wang Z., Li F., Erick Huang J., Hung S.-F., Wang Y., Li J., Nam D.-H., Li Y. C., Xu Y., Lu R., Zhang S., Lum Y., Ren Y., Fan L., Wang F., Li H.-h., Appadoo D., Dinh C.-T., Liu Y., Chen B., Wicks J., Chen H., Sinton D., Sargent E. H. (2023). Adv. Mater..

[cit54] Zhang X., Feng Y., Tang S., Feng W. (2010). Carbon.

[cit55] Choi J., Wagner P., Jalili R., Kim J., MacFarlane D. R., Wallace G. G., Officer D. L. (2018). Adv. Energy Mater..

[cit56] Zhu M., Ye R., Jin K., Lazouski N., Manthiram K. (2018). ACS Energy Lett..

[cit57] Sisler J., Khan S., Ip A. H., Schreiber M. W., Jaffer S. A., Bobicki E. R., Dinh C.-T., Sargent E. H. (2021). ACS Energy Lett..

[cit58] Mondal S., Pain T., Sahu K., Kar S. (2021). ACS Omega.

[cit59] Lu X., Wu Y., Yuan X., Huang L., Wu Z., Xuan J., Wang Y., Wang H. (2018). ACS Energy Lett..

[cit60] Ren S., Joulié D., Salvatore D., Torbensen K., Wang M., Robert M., Berlinguette C. P. (2019). Science.

[cit61] Wang M., Torbensen K., Salvatore D., Ren S., Joulié D., Dumoulin F., Mendoza D., Lassalle-Kaiser B., Işci U., Berlinguette C. P., Robert M. (2019). Nat. Commun..

[cit62] Torbensen K., Han C., Boudy B., von Wolff N., Bertail C., Braun W., Robert M. (2020). Chem. – Eur. J..

[cit63] Jiang Z., Zhang Z., Li H., Tang Y., Yuan Y., Zao J., Zheng H., Liang Y. (2023). Adv. Energy Mater..

[cit64] Monteiro M. C. O., Dattila F., Hagedoorn B., García-Muelas R., López N., Koper M. T. M. (2021). Nat. Catal..

[cit65] Li F., Li Y. C., Wang Z., Li J., Nam D.-H., Lum Y., Luo M., Wang X., Ozden A., Hung S.-F., Chen B., Wang Y., Wicks J., Xu Y., Li Y., Gabardo C. M., Dinh C.-T., Wang Y., Zhuang T.-T., Sinton D., Sargent E. H. (2020). Nat. Catal..

[cit66] Lin L., Liu T., Xiao J., Li H., Wei P., Gao D., Nan B., Si R., Wang G., Bao X. (2020). Angew. Chem., Int. Ed..

[cit67] Wu Z.-Y., Zhu P., Cullen D. A., Hu Y., Yan Q.-Q., Shen S.-C., Chen F.-Y., Yu H., Shakouri M., Arregui-Mena J. D., Ziabari A., Paterson A. R., Liang H.-W., Wang H. (2022). Nat. Synth..

[cit68] Yang H., Lin Q., Zhang C., Yu X., Cheng Z., Li G., Hu Q., Ren X., Zhang Q., Liu J., He C. (2020). Nat. Commun..

[cit69] Zhang C., Shahcheraghi L., Ismail F., Eraky H., Yuan H., Hitchcock A. P., Higgins D. (2022). ACS Catal..

[cit70] Teindl K., Patrick B. O., Nichols E. M. (2023). J. Am. Chem. Soc..

[cit71] Azcarate I., Costentin C., Robert M., Savéant J.-M. (2016). J. Am. Chem. Soc..

[cit72] Nichols E. M., Derrick J. S., Nistanaki S. K., Smith P. T., Chang C. J. (2018). Chem. Sci..

[cit73] Krisch D., Sun H., Pellumbi K., Faust K., Apfel U.-P., Schöfberger W. (2022). Catalysts.

[cit74] Siritanaratkul B., Forster M., Greenwell F., Sharma P. K., Yu E. H., Cowan A. J. (2022). J. Am. Chem. Soc..

[cit75] Eagle C., Neri G., Piercy V. L., Younis K., Siritanaratkul B., Cowan A. J. (2023). Sustainable Energy Fuels.

[cit76] Zhao Y., Wang S., Zhu L.-J., Sun M.-J., Zhang T., Cao R. (2022). ChemElectroChem.

[cit77] Creissen C. E., Fontecave M. (2022). Nat. Commun..

[cit78] McKee M., Kutter M., Lentz D., Kuehnel M., Kornienko N. (2023). ChemRxiv.

[cit79] Schild J., Reuillard B., Morozan A., Chenevier P., Gravel E., Doris E., Artero V. (2021). J. Am. Chem. Soc..

[cit80] Guo Y., Shi W., Yang H., He Q., Zeng Z., Ye J.-y., He X., Huang R., Wang C., Lin W. (2019). J. Am. Chem. Soc..

[cit81] Shimoni R., Shi Z., Binyamin S., Yang Y., Liberman I., Ifraemov R., Mukhopadhyay S., Zhang L., Hod I. (2022). Angew. Chem., Int. Ed..

[cit82] Liu Y., McCrory C. C. L. (2019). Nat. Commun..

[cit83] Nam D.-H., Shekhah O., Ozden A., McCallum C., Li F., Wang X., Lum Y., Lee T., Li J., Wicks J., Johnston A., Sinton D., Eddaoudi M., Sargent E. H. (2022). Adv. Mater..

[cit84] Li C., Qi A., Ling Y., Tao Y., Zhang Y.-B., Li T. (2023). Sci. Adv..

[cit85] Gu H., Shi G., Zhong L., Liu L., Zhang H., Yang C., Yu K., Zhu C., Li J., Zhang S., Chen C., Han Y., Li S., Zhang L. (2022). J. Am. Chem. Soc..

[cit86] Fang M., Xu L., Zhang H., Zhu Y., Wong W.-Y. (2022). J. Am. Chem. Soc..

[cit87] Al-Attas T. A., Marei N. N., Yong X., Yasri N. G., Thangadurai V., Shimizu G., Siahrostami S., Kibria M. G. (2021). ACS Catal..

[cit88] Kuruvinashetti K., Kornienko N. (2022). ChemElectroChem.

[cit89] Nitopi S., Bertheussen E., Scott S. B., Liu X., Engstfeld A. K., Horch S., Seger B., Stephens I. E., Chan K., Hahn C. (2019). Chem. Rev..

[cit90] Smith M. R., Gilman A., Hullfish C. W., Niu W., Zheng Y., Koel B. E., Sarazen M. L. (2022). J. Phys. Chem. C.

[cit91] Kim M. K., Kim H. J., Lim H., Kwon Y., Jeong H. M. (2019). Electrochim. Acta.

[cit92] Zhu Q., Yang D., Liu H., Sun X., Chen C., Bi J., Liu J., Wu H., Han B. (2020). Angew. Chem., Int. Ed..

[cit93] Wen C. F., Zhou M., Liu P. F., Liu Y., Wu X., Mao F., Dai S., Xu B., Wang X. L., Jiang Z., Hu P., Yang S., Wang H. F., Yang H. G. (2022). Angew. Chem., Int. Ed..

[cit94] Dong L.-Z., Lu Y.-F., Wang R., Zhou J., Zhang Y., Zhang L., Liu J., Li S.-L., Lan Y.-Q. (2022). Nano Res..

[cit95] Zhang Y., Dong L.-Z., Li S., Huang X., Chang J.-N., Wang J.-H., Zhou J., Li S.-L., Lan Y.-Q. (2021). Nat. Commun..

[cit96] Gui J., Li L., Yu B., Wang D., Yang B., Gu Q., Zhao Y., Zhu Y., Zhang Y. (2023). ACS Appl. Mater. Interfaces.

[cit97] Zhang F., Wang P., Zhao R., Wang Y., Wang J., Han B., Liu Z. (2022). ChemSusChem.

[cit98] Nam D.-H., De Luna P., Rosas-Hernández A., Thevenon A., Li F., Agapie T., Peters J. C., Shekhah O., Eddaoudi M., Sargent E. H. (2020). Nat. Mater..

[cit99] Li J., Zhang Y., Kornienko N. (2020). New J. Chem..

[cit100] Chen J.-M., Xie W.-J., Yang Z.-W., He L.-N. (2022). ChemSusChem.

[cit101] Harmon N.
J., Wang H. (2022). Angew. Chem., Int. Ed..

[cit102] Xu Y., Edwards J. P., Zhong J., O'Brien C. P., Gabardo C. M., McCallum C., Li J., Dinh C.-T., Sargent E. H., Sinton D. (2020). Energy Environ. Sci..

[cit103] Luc W., Ko B. H., Kattel S., Li S., Su D., Chen J. G., Jiao F. (2019). J. Am. Chem. Soc..

[cit104] Komatsu S., Tanaka M., Okumura A., Kungi A. (1995). Electrochim. Acta.

[cit105] Ko B. H., Hasa B., Shin H., Jeng E., Overa S., Chen W., Jiao F. (2020). Nat. Commun..

[cit106] Legrand U., Apfel U. P., Boffito D. C., Tavares J. R. (2020). J. CO2 Util..

[cit107] Lu X., Jiang Z., Yuan X., Wu Y., Malpass-Evans R., Zhong Y., Liang Y., McKeown N. B., Wang H. (2019). Sci. Bull..

[cit108] Li P., Lu X., Wu Z., Wu Y., Malpass-Evans R., McKeown N. B., Sun X., Wang H. (2020). Angew. Chem., Int. Ed..

[cit109] Jackson M. N., Oh S., Kaminsky C. J., Chu S. B., Zhang G., Miller J. T., Surendranath Y. (2018). J. Am. Chem. Soc..

[cit110] Kaminsky C. J., Weng S., Wright J., Surendranath Y. (2022). Nat. Catal..

[cit111] Abdinejad M. , Farzi A., Möller-Gulland R., Mulder F., Liu C., Shao J., Robert M., Seifitokaldani A. and Burdyny T., Eliminating redox-mediated electron transfer mechanisms on molecular catalysts enables CO_2_ conversion to ethanol, Research Square, 2023

[cit112] Rao H., Schmidt L. C., Bonin J., Robert M. (2017). Nature.

[cit113] Boutin E., Wang M., Lin J. C., Mesnage M., Mendoza D., Lassalle-Kaiser B., Hahn C., Jaramillo T. F., Robert M. (2019). Angew. Chem., Int. Ed..

[cit114] Ren X., Zhao J., Li X., Shao J., Pan B., Salamé A., Boutin E., Groizard T., Wang S., Ding J., Zhang X., Huang W.-Y., Zeng W.-J., Liu C., Li Y., Hung S.-F., Huang Y., Robert M., Liu B. (2023). Nat. Commun..

[cit115] Li J., Zhang Y., Kuruvinashetti K., Kornienko N. (2022). Nat. Rev. Chem.

[cit116] Li J., Al-Mahayni H., Chartrand D., Seifitokaldani A., Kornienko N. (2023). Nat. Synth..

[cit117] Zheng W., Lee L. Y. S. (2021). ACS Energy Lett..

[cit118] Zhang J., My Pham T. H., Gao Z., Li M., Ko Y., Lombardo L., Zhao W., Luo W., Züttel A. (2023). ACS Catal..

[cit119] Lamagni P., Miola M., Catalano J., Hvid M. S., Mamakhel M. A. H., Christensen M., Madsen M. R., Jeppesen H. S., Hu X.-M., Daasbjerg K., Skrydstrup T., Lock N. (2020). Adv. Funct. Mater..

